# SHH Signaling Pathway Drives Pediatric Bone Sarcoma Progression

**DOI:** 10.3390/cells9030536

**Published:** 2020-02-26

**Authors:** Frédéric Lézot, Isabelle Corre, Sarah Morice, Françoise Rédini, Franck Verrecchia

**Affiliations:** INSERM UMR1238, PHY-OS, “Bone sarcomas and remodeling of calcified tissues”, Nantes University, 44000 Nantes, France; frederic.lezot@univ-nantes.fr (F.L.); isabelle.corre@univ-nantes.fr (I.C.); sarah.morice@univ-nantes.fr (S.M.); francoise.redini@univ-nantes.fr (F.R.)

**Keywords:** Sonic Hedgehog, Gli, skeletal development, osteosarcoma, Ewing sarcoma

## Abstract

Primary bone tumors can be divided into two classes, benign and malignant. Among the latter group, osteosarcoma and Ewing sarcoma are the most prevalent malignant primary bone tumors in children and adolescents. Despite intensive efforts to improve treatments, almost 40% of patients succumb to the disease. Specifically, the clinical outcome for metastatic osteosarcoma or Ewing sarcoma remains poor; less than 30% of patients who present metastases will survive 5 years after initial diagnosis. One common and specific point of these bone tumors is their ability to deregulate bone homeostasis and remodeling and divert them to their benefit. Over the past years, considerable interest in the Sonic Hedgehog (SHH) pathway has taken place within the cancer research community. The activation of this SHH cascade can be done through different ways and, schematically, two pathways can be described, the canonical and the non-canonical. This review discusses the current knowledge about the involvement of the SHH signaling pathway in skeletal development, pediatric bone sarcoma progression and the related therapeutic options that may be possible for these tumors.

## 1. Introduction: Osteosarcoma and Ewing Sarcoma 

Osteosarcoma (OS) and Ewing sarcoma (ES) are the two most common malignant primary bone tumors observed in children, adolescents and young adults, occurring primarily in the second decade of life (median age occurrence of 18 and 15 years, respectively, for OS and ES). They are qualified as rare tumors as they represent about 6% of all childhood malignancies and less than 0.2% of all cancers, whatever the age. 

Osteosarcoma is the most frequent type of malignant primary tumor of the bone, with an incidence of three in one million. Boys are slightly more affected than girls. About 80% of osteosarcoma occur at the metaphysis of long bones, with the most common sites being the distal femur, the proximal tibia and the proximal humerus. Conventional OS, defined by cells that produce varying amounts of osteoid matrix (mineralized or not), is the most common histologic subtype, accounting for approximately 75% of all cases [1,2,3]. It is then divided in three major histologic subtypes: osteoblastic (50%), chondroblastic (25%) and fibroblastic (25%) OS. This rare cancer (around 600 cases per year in Europe) presents radiographically-mixed lesions with lytic and sclerotic territories, in variable proportion depending on the tumors [4]. Whole-genome sequencing analyses have shown that OS displays high rates of genetic alterations, containing many somatic mutations and copy number alterations. TP53 and RB1 show recurrent somatic alterations in concordant studies, suggesting that they could be key players in bone oncogenesis [5].

Ewing sarcoma is the second most common malignant primary bone tumor in children, adolescents and young adults. The annual incidence is estimated at 1–3 in one million. Staging procedures identify about 25% of patients that have metastases at diagnosis. Typically, ES arises in bone sites, with the most common sites including the long bones (47%), pelvis (26%), chest wall and spine. In long bones, ES predominantly occurs at the diaphysis. The osseous mass may coexist with a soft tissue component, resulting in a Coleman triangle or a multilayered periosteal reaction. On the contrary to OS, ES can occasionally develop in soft tissues [6]. The Ewing sarcoma family is characterized as tumors consisting of small round malignant cells that may exhibit different levels of neural differentiation. Ewing sarcoma, malignant peripheral neuroectodermal tumor, Askin tumor and atypical Ewing sarcoma are now summarized under the term Ewing sarcoma. Tumor-specific, non-random gene rearrangements of chromosome 22 are found in more than 95% cases. The most common rearrangement is the t(11 :22)(q24 :q12) translocation, found in 85% of these tumors [7]. 

## 2. Current Main Treatments and Perspectives

Significant improvement in overall survival has been achieved with the use of multi-agent chemotherapy in conjunction with local therapy approaches, namely surgery and radiotherapy in the case of Ewing sarcoma. 

However, despite multi-agent chemotherapy and aggressive surgical resection sparing the limb, 30% of OS patients with localized disease and 80% with metastatic disease at diagnosis will relapse [8]. Standard therapy consists of the surgical removal of any resectable primary tumor and metastases, combined with neoadjuvant and adjuvant regimens of multi-agent chemotherapy, including four agents: doxorubicin (adriamycin), cisplatin, high-dose methotrexate and ifosfamide for patients with high-risk or metastatic disease. Surgery is the only adequate available treatment option to achieve local control in osteosarcoma. Indeed, due to the low radiosensitivity of osteosarcoma, radiotherapy does not play a major role in the multimodal treatment. However, there may be some situations where its use may be beneficial, for example, when primary lesions are not accessible for surgery. This modern multimodal therapy yields 75% survival at 5 years for osteosarcoma patients without overt metastasis at diagnosis, but the outcome for metastatic, relapsed disease (locally or distal) or for patients not responding to chemotherapy remains poor, at less than 30% survival at 5 years.

Before the era of combination chemotherapy, the prognosis of ES patients was poor, with more than 90% of patients dying from secondary metastases [9]. The current standard of care for ES consists mainly of chemotherapy with 5 drugs (vincristine/doxorubicin/cyclophosphamide alternating with ifosfamide/etoposide) and associated with local surgery or radiotherapy when surgery is difficult [10]. While this intensive multi-agent chemotherapy has improved survival compared with the pre-chemotherapy era, there have been few recent improvements in the outcome for ES patients with either localized disease or those who present with metastatic disease at diagnosis. In addition, it has been difficult even for therapies that prove to be beneficial, such as mifamurtide in osteosarcoma, to obtain regulatory approval [11]. 

However, great advances have been made recently in understanding the molecular basis of pathogenesis and the progression of OS and ES. This new understanding has been achieved in parallel with an explosion of novel therapies developed to specifically inhibit cancer-associated genes and pathways. Identification of key regulatory pathways and molecular biomarkers has yielded dramatic changes in the outcome for several adult cancers, but childhood cancers, in general, and bone sarcoma, in particular, have been largely sidelined in this revolution.

To help make these important discoveries relevant for pediatric bone sarcoma, it is important to have an understanding of the role of each signaling pathway in the biology of the disease as well as of the available agents that target these processes. Priority was given to the pathways for which there is good information available about the relevance to OS and ES. The main novel therapies that can be applied in children’s bone sarcomas involve (i) surface markers for OS (receptor tyrosine kinases, such as insulin-like growth factor receptor type I, human epidermal growth factor receptor 2 and the ERBB family, platelet-derived growth factor receptor), (ii) intracellular signaling pathways (Ezrin, mammalian target of rapamycin, steroid receptor co-activator, Notch, Hedgehog, histone deacetylase inhibitors, Ras, MDM2), (iii) targeting bone metabolism (bisphosphonates, conjugated radioisotopes, denosumab), and (iv) environmental and immune interactions of bone sarcoma (immunotherapy: mifamurtide, sargramostim, other immunomodulators, such as interferon-γ, other immunotherapies including tumor vaccine, targeted therapy using antibodies for GD2 tumor antigens). Immunotherapy approaches are promising for pediatric sarcoma treatment. Environmental interactions involving the matrix and vasculature are also attracting. Part of the pathogenesis of bone sarcomas includes the ability to invade through extracellular matrix tissues and to recruit a new blood supply as tumors grow and disseminate [12]. These activities proceed by hijacking normal biological processes that are then exploited by tumor cells to facilitate their growth and spread [13]. Inhibition of MMP2 and MMP9 affects OS tumor growth and metastasis formation [14,15]. Preclinical efficacy of VEGF-based therapeutics, including anti-VEGF antibodies and small molecule inhibitors against VEGFR, have been confirmed in pediatric bone sarcomas [16]. Very recently, a strategy using regorafenib, a multi-kinase inhibitor of angiogenic (VEGF-R1-3, TIE-2), stromal (PDGFR-β, FGFR) and oncogenic kinases (KIT, RET, and RAF), was tested. Two French and American clinical trials were conducted in patients with recurrent, progressive, metastatic OS after failure of conventional chemotherapy and they demonstrated a significant extension of progression-free survival in both trials [17,18].

## 3. SHH Signaling Pathway

**Figure 1:**
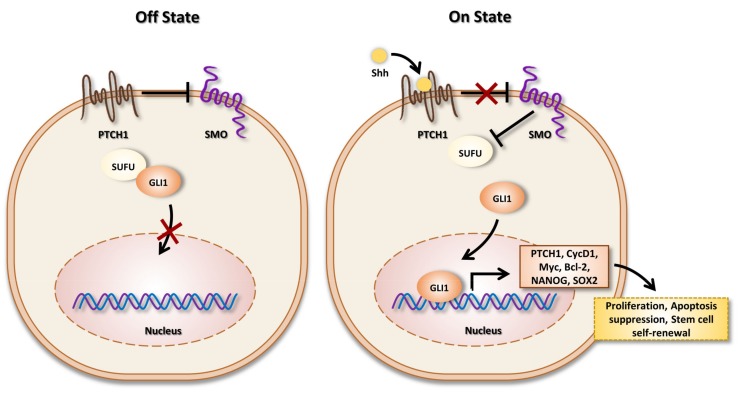
Off state (left): In the absence of Sonic Hedgehog (SHH) ligand, Ptch inhibits signal transduction by SMO. Gli canonical SHH signaling pathway proteins are sequestered in the cytoplasm by interaction with Sufu. Therefore, the expression of SHH signaling targets turns off. On state (right): In the presence of ligand, SHH binds to Ptch and, therefore, induces the translocation of SMO to the primary cilium, a subcellular compartment essential for signal transduction to the positive forms of Gli proteins. Gli proteins accumulate into the nucleus and activate target genes transcription.

The Hedgehog (HH) signaling pathway is a major evolutionarily conserved pathway that regulates key events during the development process and tissue homeostasis, such as bone [19]. The HH gene was discovered in 1980 by Nusslein-Volhard and Wieschaus through the analysis of *Drosophila melanogaster* [20]. In the early of 1990s, three HH ligands were described in vertebrates: Sonic, Indian and Desert Hedgehog, differentially expressed depending on the animal species. In humans, Sonic Hedgehog (SHH), the main ligand, is produced and transported into the endoplasmic reticulum (ER) and Golgi apparatus in which it undergoes autoprocessing [21]. SHH, initially synthetized as a 45kDa precursor, is thus cleaved into a 19 kDa (SHH-N) and a 26 kDa (SHH-C) secreted peptide. SHH-N is modified by the addition of lipid (palmitoyl and cholesterol) and mediates signaling in vertebrates and invertebrates [22]. SHH-C is believed to possess any biological activity under physiological condition [23]. Of note, SHH at the cell surface can be released by lipid bilayer membrane vesicles, called exosomes [24,25]. The activation of the SHH cascade can be done through different pathways: schematically, two pathways can be described, the canonical (Figure 1) and the non-canonical.

The canonical cascade is based on the binding of SHH on the 12-transmembrane receptor Patch (Ptch) [26], which, in the absence of ligand, continuously inhibits the activity of the G-coupled receptor Smoothened (SMO). In the presence of ligand, Ptch undergoes endocytosis, which leads to its degradation in the lysosome [27], resulting in the activation of SMO. Suppressor of fused homolog (Sufu) is a crucial inhibitor of the SHH cascade [28]. Indeed, Sufu sequesters glioma-associated oncogene homolog (Gli) in the cytosol [29]. The mammalian Gli gene family comprises three isoforms: Gli1, Gli2 and Gli3 (Figure 1). Of note, the human Gli1 gene was first identified by Volgestein and colleagues as a putative oncogene amplified in glioblastoma [30]. SMO activation generates an intracellular signal that induces the dissociation of the Gli-Sufu complex and promotes the translocation of Gli proteins in the nucleus where they act as transcriptional factors by binding to a consensus sequence of 5’-GACCACCCA-3’ [31,32] through the DNA-binding domain, which consists of five C2H2-Kruppel-type zinc-finger motifs [33,34]. The transcriptional activity of Gli promotes the expression of various genes among them: SHH cascade compounds, such as Gli1 and Ptch1, pro-proliferation genes, such as Cyclin D1 and Myc, or cell cycle regulators, such as CCND2 and CCNE1 [35].

The non-canonical cascade exists to elicit various cellular responses, especially in cancers [36]. One of the mechanisms of non-canonical SHH signaling involves the activation of Gli transcriptional factors independently of SHH, Ptch and SMO. For example, epidermal growth factor receptor (EGFR) can induce the activation of Gli through the extracellular signal-regulated kinase (ERK) pathway during oncogenic transformation [37]. Ras signaling induces Gli1-transcription in gastric cancer [38]. The TGF-β cascade can also enhance Gli expression, especially Gli2, via Smad3-dependant mechanisms in melanoma [39,40,41]. Regarding bone sarcoma, it has been established that Gli1 is a direct transcriptional target of EWS-Fli1 in Ewing Sarcoma [42].

## 4. SHH and Skeletal Development

**Figure 2:**
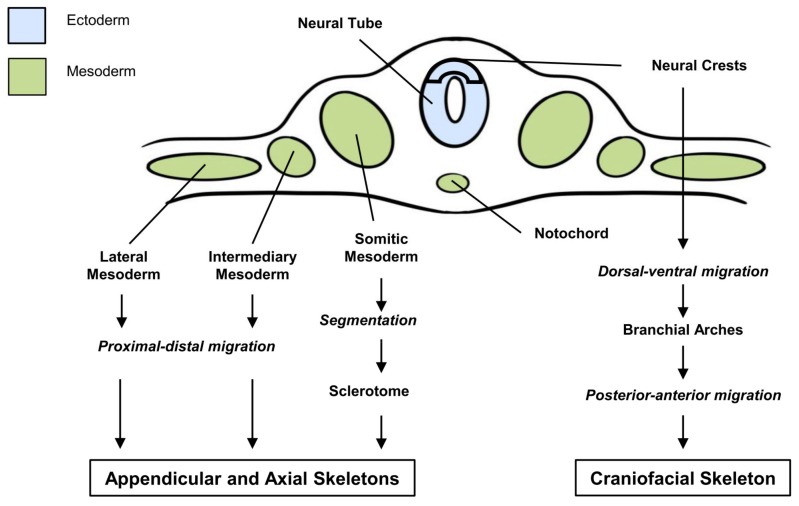
Origins of appendicular, axial and craniofacial skeletons. The axial and appendicular skeletons derive from cells of the paraxial mesoderm following a proximal-distal migration of cells from the lateral and intermediary mesoderm or the segmentation of cells from the somatic mesoderm. The craniofacial skeleton derives from the ectoderm cells of the neural crests following a dorsal-ventral migration, enabling the formation of the branchial arches and a posterior-anterior migration.

The skeleton is composed of five different mineralized tissues. The bone and cartilage tissues constitute the main part of the skeleton and the enamel, dentin and cementum are dental-specific tissues. Embryologically, the cells forming these tissues derive from the neural crest for the craniofacial skeleton (most skull bones and teeth) and from the paraxial (somatic, intermediary and lateral) mesoderm for the axial and appendicular skeletons (Figure 2). SHH was shown to be a crucial factor for the migration and the predetermination of progenitor cells for skeletal tissues that originated from either the neural crests or mesoderm [43,44,45,46,47,48,49,50,51]. Indeed, any perturbation of SHH expression/function during these early steps of embryonic development leads to severe alterations of the skeleton development with reduction in the size or absence of certain elements of the craniofacial, axial or appendicular skeletons [49,52,53,54,55,56,57,58,59,60,61,62,63,64]. Such alterations of SHH, whatever their causes, are often lethal before birth as they are also associated with the defective formation of important none-skeletal organs.

During the following embryonic steps of skeleton formation, corresponding to the morphogenesis of the different skeletal elements, which is governed by numerous sequential interactions between tissues (epithelial-mesenchymal, neuro-mesenchymal, etc.), SHH was shown to be an important morphogen [49,65,66,67,68,69]. Indeed, SHH expression was associated with different organization centers participating with other factors of the FGF, BMP and WNT families to determine morphogenic fields.

**Figure 3:**
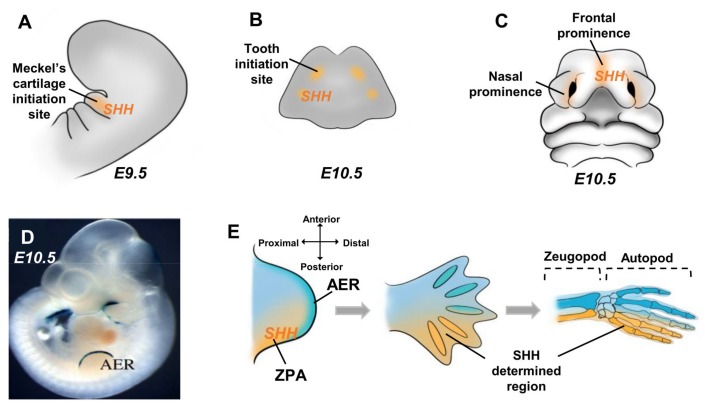
Sonic Hedgehog (SHH) implications in the craniofacial and limb morphogenesis. (**A**) SHH expression pattern in the first branchial arch determines the Meckel cartilage initiation site on mouse embryonic day 9.5 (E9.5). (**B**) SHH expression on mouse embryonic day 10.5 mandible determines the tooth initiation site. (**C**) SHH expression in the frontonasal prominences of the mouse embryonic day 10.5 governs the morphogenesis of the upper facial structures. (**D**–**E**) In the mouse limb bud at embryonic day 10.5, SHH expression in the zone of polarizing activity (ZPA) determines the posterior region of the limb bud, where distal growth is controlled by the apical ectodermal ridge (AER) visualized by Dlx2/LacZ expression in (**D**) and the subjacent progression zone. The cells determined by the SHH signaling will give rise to the posterior bones of the autopod and zeugopod (**E**).

In the craniofacial skeleton, SHH is expressed in the first branchial arch [49,70], participating successively to determine Meckel’s cartilage formation [45,71] (Figure 3A) and the dental regions (Figure 3B) [72,73,74,75,76,77] in the frontonasal prominences [78] that govern the formation of the different upper facial structures (Figure 3C) and in the cranium sutures that control the harmonious growth of the different bones of the skull with the underlying forebrain [79,80,81,82].

In the appendicular skeleton, SHH is implicated in the morphogenesis of the limb [83,84], which can be subdivided into three stages called the bud, the pallet and the rotations. The limb buds appear as expansions of the lateral mesoderm covered by the ectoderm (Figure 3D). A thickening of the ectoderm at the distal end of each bud forms the apical ectodermal ridge (AER). This ridge plays a fundamental role in elongating the bud (Figure 3E). The underlying mesenchyme is called the progression zone (Figure 3E). Another important mesenchymal area in the posterior part of the bud is called the zone of polarizing activity (ZPA) that determines the developing bud along the anterior/posterior axis (Figure 3E). SHH is expressed in the ZPA [85], whose function is the early determination of the proximal-distal identity of the mesenchymal cells from the limb bud [86,87,88,89,90] with an important consequence for the autopod and zeugopod polarization (Figure 3E). The pallet stage is characterized by the rapid elongation of the bud and the formation in its distal part of a flattened structure: the pallet. As the pallet grows, inter-digital furrows appear, resulting from the apoptosis of mesoderm cells that separate pre-cartilage blanks from fingers and toes. The digits that form in the posterior region are known to be under the influence of SHH [89,91,92,93]. Any perturbations in the SHH expression level or pattern during the bud and pallet stages of limb morphogenesis, whatever their genetic or environmental origins, lead to a large spectrum of limb dysmorphic features ranging from the absence of most of the autopod to polydactylism [94,95,96,97]. During the last stage of the limb morphogenesis, the rotation stage, the arm and leg blanks rotate so that the elbow is laterally oriented and the knee is ventral. To date, SHH has not been implicated in the rotation stage.

In the axial skeleton, which derives from the somitic mesoderm (Figure 2), SHH also plays a major morphogenetic role. Embryonic precursors of cartilage in the ribs and vertebrae are localized within the somite. Somites are paired segmented structures that form epithelial spheres from the ventral region, which cells leave to form the sclerotome. The ventral portion of the sclerotome surrounds the notochord and forms the rudiment of the vertebral body while the dorsal portion surrounds the neural tube and forms the rudiment of the vertebral arches. SHH, produced by the notochord, was shown as the primary signal for sclerotome induction. In cases of SHH expression disruptions in the notochord, the axial skeleton formation is always severely affected with presence of caudal dysgenesis, cyclopia and rib absence [98,99,100,101,102].

After morphogenesis, the organogenesis of the skeletal elements is initiated. SHH was implicated in both tooth and bone organogenesis. Concerning the tooth, SHH was expressed by the main organization centers, called enamel knocks, that control the proliferation and functional differentiation of the cells secreting the enamel and the dentine, respectively called the ameloblast and odontoblast. Defects in SHH expression in the enamel knocks lead to severe alterations of the size and the shape of the teeth and to disruptions of the polarity and organization of the ameloblast and odontoblast layers [75]. Regarding bone organogenesis, three bone formation processes exist: the endochondral, the intramembranous and the periosteal apposition. Endochondral bone formation is the process by which growing cartilage in the extremities of long bone is systematically replaced by bone. This process is implicated in the growth in the length of long bone. The periosteal apposition enables the growth in the width of the long bone and is secondary to the endochondral bone formation. The membranous bone formation, which is implicated in the formation of flat bones (mainly in the skull), is initiated by a condensation of mesenchymal stem cells that gradually differentiate into osteoprogenitor then osteoblast cells and directly secrete a bone matrix without requiring a cartilaginous phase.

Regarding endochondral bone formation, SHH has not been directly implicated, in contrast to IHH (Indian Hedgehog), which plays a major role in the differentiation of chondroblast cells of the growth plate [103,104,105]. In intramembranous bone formation and the periosteal apposition, several studies have established that SHH can modulate the differentiation and activation of osteoblastic cells via its expression in the primary cilia [106,107,108]. Interestingly, studies focusing on the healing and/or regeneration of bone and cartilage tissues have established that SHH was able to improve the neo-differentiation of osteoblasts and chondroblasts with an interesting therapeutic potential [109,110,111,112,113].

## 5. SHH Signaling in OS et ES

Beside its critical role in multiple developmental processes of organs and tissues, such as bone (see above), the Hedgehog-Gli signaling pathway is aberrantly activated in several human cancers [35,36,114,115,116,117,118].

Mutations in some of the genes involved in the SHH cascade, such as SMO, Ptch or Sufu, have been associated with the development of cancers [119,120,121]. For example, mutations of Ptch were originally described in Gorlin syndrome, a rare hereditary disease with an autosomal dominant transmission, characterized by the association of multiple basal cell carcinoma, medulloblastoma and rhabdomyosarcoma [122,123]. Sporadic mutations of SHH have been described in basal cell carcinoma, medulloblastoma and in breast carcinoma cell lines [117,124]. 

The SHH pathway can be also activated without mutations of the genes involved in the SHH cascade, mainly through a paracrine effect of SHH and, thus, on the canonical cascade [35,36,115,116,117,118]. Schematically, Gli transcriptional factor may act at different steps of tumorigenesis [118]. The main properties of Gli family proteins are to stimulate cell proliferation and tumor growth in many cancers [35,36,115,116,117,118]. The SHH cascade was also implicated in the ability of cells to (i) acquire a migratory phenotype and (ii) migrate, invade adjacent tissue and, thus, to metastasis [35,36,115,116,117,118]. 

In the context of bone tumors, the relevance of the HH pathway emerged progressively in the last decade in osteosarcoma (OS) and Ewing sarcoma bone tumors. Several preclinical studies highlighted the overexpression of HH components in OS cell lines. Ligands SHH, DHH and IHH, Receptors Ptch1 and SMO, and transcription factors Gli1 and Gli2 are significantly overexpressed in human osteosarcoma cell lines, both at the mRNA and protein level [125]. Importantly, these findings were also validated in OS tumor patients; several studies investigated the HH pathway on human samples mainly by RT-PCR analysis of HH component genes. In a small cohort of 12 patients at diagnosis, expression of *SMO, PTCH1* and *Gli2* transcripts were variously increased [126]. Recently, an exploratory study of surgical biopsies of 43 high-grade OS also revealed high gene expression of *IHH, PTCH1* and *Gli1*, and moderated expression of *SMO*, normalized to normal osteoblast samples [127]. The levels of IHH, *SMO, PTCH1* and *Gli1* were correlated with each other in small tumors, indicative of a ligand-independent activation whereas ligand-dependent activation of the HH pathway was suggested in large tumors, as high expression of *IHH* and *PTCH1* were correlated. Importantly, this study aimed at evaluating the prognostic value of the expression of the HH pathway and, despite no statistically differences due to the limited sample size, suggested that the expression of *IHH* could be predictive of outcome in combination with tumor size, as patients with lower *IHH* expression and small tumors had better survival compared to patients with high *IHH* expression and large tumors. A positive correlation was also identified between *Gli1* expression and chemotherapy-induced necrosis, indicative of increased responsiveness to treatment in patients stratified into the high *Gli1* expression group. Furthermore, Yang et al. [128] showed, in an immunohistochemistry study on a tissue-microarray of 58 OS samples, that overall survival was shortened in patients with high *Gli2* expression and that, during the 5 years of follow-up, living patients significantly expressed lower *Gli2* compared to patients who died. Altogether, these results suggested that expression of HH signaling components might be useful as a prognostic but also as a theranostic factor. 

The functional significance of altered HH signaling in OS has been deciphered in several models of cell lines and in preclinical animal models. SMO-dependent HH signaling regulates the cell cycle in OS, favoring progression into G1 through regulation of cyclin D1 and E1. Moreover, inhibition of SMO by cyclopamine, a specific inhibitor of SMO, or by genetic invalidation of SMO, prevented cell proliferation in vitro and tumor growth in vivo [126]. Additional studies focused on the role of the transcription factor Gli2 in OS development and progression. In SaOS2 and 143B OS cell lines, inhibition of Gli2 prevented OS proliferation in vitro, through inhibition of cell cycle proteins cyclin D1, pRb and SKP2 [129]. Importantly, overexpression of potent activated Gli2 in immortalized human mesenchymal stem cells stimulated their proliferation, reinforcing a role for Gli2 in osteosarcoma genesis as osteosarcoma cells are of mesenchymal origin. In addition, invalidation of Gli2 in KHOS and U2OS cell lines has been shown to induce a decrease in OS proliferation both in 2D and 3D cultures and to promote OS cell sensitivity to chemotherapies doxorubicin and methotrexate [128]. Use of GANT61, a Gli1/2 antagonist, also displayed an inhibitory effect on OS cell lines [130]. Several strategies targeting the HH signaling pathway in preclinical animal models have reinforced the relevance of this pathway in OS. In a 143B-induced xenograft model, targeting of Gli2 by genetic invalidation in tumor cells inhibited tumor growth and provided a significant survival benefit [129]. In patient-derived xenograft (PDX) models, Lo et al. treated mice orally with IPI-926, a semi-synthetic analog of cyclopamin and small-molecule inhibitor of SMO, and showed a specific inhibition of ligand-dependent HH pathway and a significant decrease in tumor weight and volume [130]. Targeting Gli proteins with arsenic trioxide (ATO) in a nude mouse model of subcutaneously-grafted 143B OS cells also prevented OS growth [131]. Arsenic trioxide was initially described as a potent antagonist of the HH pathway by preventing the accumulation of Gli2 in primary cilia by reducing its stability [132]. However, this chemical was also identified as an inhibitor of the transcriptional activity of Gli1/-2 [133] in Ewing sarcoma. In OS cell lines, ATO promotes apoptotic cell death and, importantly, induces the accumulation of DNA damage [131]. This latter result suggests that activated HH signaling pathways could protect OS cancer cells from DNA damage-induced death. This type of death is required for an optimal efficiency of radiotherapy or chemotherapy. Therefore, cancer cells harboring activated HH pathways could present some level of radio-resistance. In fact, Chen et al. demonstrated that activation of the HH pathway protected human hepatocellular carcinoma cells from ionizing radiation through an unclear mechanism of impaired DNA damage repair machinery [134]. Importantly, this radioprotection was alleviated by SHH antibody neutralization or Gli1 invalidation. Very recently, activation of SHH signaling was found to be involved in the resistance of OS cells to ionizing radiations [135] as radio-resistant OS MG63 cells expressed increased level of SHH, SMO, Ptch1 proteins and increased nuclear localization of Gli1 compared to radiosensitive cells, whereas silencing of SHH rendered the radio-resistant cells more radiosensitive [136]. In summary, the targeting of HH signaling appears to be an important strategy to consider in OS treatment, both for direct anti-tumoral action and also for a potentiation of the therapeutic effects of chemotherapy and/or radiotherapy.

Mechanisms of Gli2 overexpression in OS are still unclear. Both ligand-dependent and ligand-independent activation of HH have been highlighted in OS cells [130]. Moreover, the exclusive expression of SHH in the stroma compartment in a patient-derived xenograft model suggested an active paracrine communication between tumor and stroma [130]. Indeed, a dialogue between stroma and OS cells in the activation of the HH signaling pathway has been recently identified as extracellular vesicles (EVs) isolated from MSC stimulated the expression of Ptch1, SHH, and Gli1 proteins in OS cells, associated with increased cell proliferation and migration [137]. A recent study also reported the decreased expression of miR-141-3p, an miR targeting Gli2 in OS cell lines and tumor samples, suggesting an miR implication in HH-altered pathways in OS [138]. 

**Figure 4:**
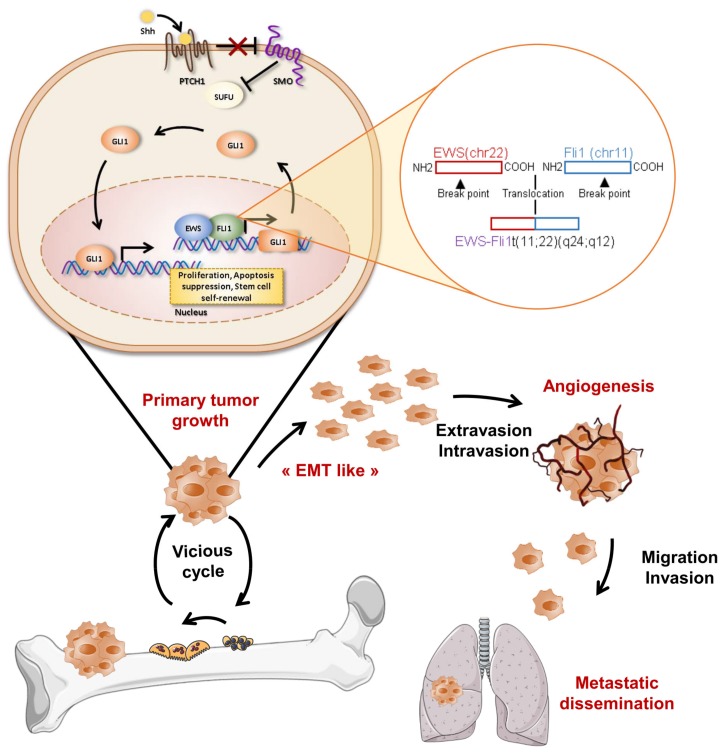
Role of SHH/Gli cascade in Ewing sarcoma development. Upper panels: EWS-Fli1 protein regulates Gli1 gene expression at the transcriptional level and, therefore, promotes the expression of Gli gene targets implicated in various cellular events, such as cell proliferation and migration. Lower panels: progression of Ewing sarcoma development. During primary tumor growth, cancer cells produce soluble factors, such as growth factors or cytokines, that activate osteoclastogenesis and, in turn, bone degradation. This bone resorption then allows the release of trapped growth factors into the bone matrix able to stimulate tumor growth, “EMT-like” (epithelial mesenchymal transition) angiogenesis and, therefore, the metastatic process.

Literature is scarce when considering the HH pathway and Ewing sarcoma (ES). Nevertheless, several genes of the HH pathway were found to be associated with metastasis in a micro-array analysis of 27 ES patients [139]. The transcription factor Gli1 appears particularly important in ES, as it is overexpressed in patient tumor specimens and in ES cell lines [140]. Gli1 was identified as a direct transcriptional target of EWS-FL1 [42] through an HH ligand-independent mechanism and appears essential in EWS-Fli1-induced tumorigenic signaling as its direct inhibition by si/sh-RNA or by chemical small-molecule inhibitor NCS75503 [42,140] impaired proliferation and clonogenicity of ES cells. Following these studies, targeting the HH/Gli1 pathway through Gli1 inhibition was envisaged in a preclinical xenograft models of ES. The injection of arsenic trioxide, the inhibitor of Gli1/2 described above, efficiently blocked ES tumor growth [133]. This initial study underlines the potential therapeutic targeting of HH/Gli1 in ES patients overexpressing Gli1 (Figure 4).

## 6. Conclusions and Clinical Perspectives

Accumulating evidence has demonstrated the rationale to target the SHH cascade in pediatric bone tumors. The SHH pathway can be blocked at different levels by several compounds that could be used as anticancer drugs. More than 50 SHH cascade inhibitors have been synthetized during the last decades [116,141,142]. The first agent was cyclopamine, a natural alkaloid derived from corn lily *Veratrum californicum*, identified as an SMO receptor inhibitor [143]. Several synthetic SMO inhibitors with a higher efficiency were then developed and tested in various cancer preclinical models and in phase I, II or III clinical trials, such as GDC-0449 (Vismodegib/Erivedge), LDE225 (Erismodegib/Sonidegib/Odomzo), IPI-926 (Saridegib), BMS-833923/XL139, PF-04449913 (Glasdegib), LY2940680 (Taladegib) or IPI-926 (Vismodegib/Erivedge) [116,144]. Most of them have been tested against carcinoma, medulloblastoma or leukemia. Interestingly, one has been tested in bone sarcoma, specifically in progressive grade 1 or 2 conventional chondrosarcoma: the GDG-0449 [145]. As reviewed above, although SMO inhibitors possess a real potential to treat cancers, many cancers have alternative mechanisms (non-canonical pathways) to activate Gli signaling through effectors that are downstream of SMO, thus rendering SMO inhibitors ineffective. In this context, several Gli inhibitors have been synthetized during the last decades, such as HPI-1, HPI-2, GANT58 or GANT61, and tested in various cancer preclinical models. Mechanistically, GANT58 and GANT61 act in the nucleus to block Gli transcriptional activity. Since they directly block Gli activity, they are expected to be active against cancers in which Gli is overexpressed, such as Ewing sarcoma.
